# Vape shops: who uses them and what do they do?

**DOI:** 10.1186/s12889-018-5467-9

**Published:** 2018-04-23

**Authors:** Julie Pattinson, Sarah Lewis, Manpreet Bains, John Britton, Tessa Langley

**Affiliations:** Division of Epidemiology and Public Health, University of Nottingham, Nottingham, UK

**Keywords:** E-cigarettes, Vape shops, Smoking cessation, Harm reduction

## Abstract

**Background:**

‘Vape shops’ are a popular source for buying electronic cigarettes (e-cigarettes) and related products. The products that vape shops sell, their marketing techniques and the extent to which they provide information or encouragement to smokers to quit tobacco use, as well as the patterns of tobacco and e-cigarette use of their customers are not well understood.

**Methods:**

We conducted cross-sectional surveys in vape shops in the East Midlands region of the United Kingdom, one with shop staff (*n* = 41), and one with customers (*n* = 197).

**Results:**

The majority of customers (84%) currently used e-cigarettes. Among current vapers, 19% were dual users and 78% had quit smoking. Over half of vapers reported using a lower level of nicotine in their current e-liquid than when they started using e-cigarettes.

There was a wide variety in products and price ranges between the shops. Many staff reported that customers ask for information about quitting smoking (90%). Less than half reported providing smoking cessation advice, although 76% of staff reported feeling confident about delivering cessation advice to customers who ask for it. Just under half of customers and shop staff said they thought it was appropriate to deliver formal in-store smoking cessation support.

**Conclusions:**

The majority of vape shop customers are vapers who have quit smoking. Shop staff play a central role in providing customers with product information, and many provide smoking cessation advice. Further research is needed to investigate the potential for smoking cessation interventions in vape shops, including the extent to which these would appeal to non-vapers.

**Electronic supplementary material:**

The online version of this article (10.1186/s12889-018-5467-9) contains supplementary material, which is available to authorized users.

## Background

Electronic cigarettes (e-cigarettes) provide a less hazardous alternative to smoked tobacco for smokers, and can aid smoking cessation [1–6]. E-cigarettes are increasingly widely used, with 20% of smokers and recent ex-smokers in England reporting use in the first quarter of 2017, compared with 3% in 2011 [7]. E-cigarettes are now the most commonly used cessation aid in England; an estimated 34% of adults who quit or made a quit attempt in the last year reported using e-cigarettes in their most recent quit attempt, compared with 21% using over-the-counter nicotine replacement therapy and less than 3% receiving behavioural support [7]. The number of specialist e-cigarette shops has also increased, with recent estimates suggesting that the UK has 1700 ‘vape shops’ [8–10]. The high street locations of vape shops make them an easily accessible option for smokers, and they are currently the most popular source for purchasing e-cigarettes in England [7].

Smokers who use vape shops are likely to be interested in temporary abstinence, cutting down or quitting smoking. Since vape shop staff have a potential major role in capitalising on opportunities to promote smoking cessation through their engagement with customers, it is important to understand the products they sell and marketing techniques they employ, and the extent to which they provide information or encouragement to smokers to quit all tobacco use. To date, the available evidence is limited to studies of vape shops in the USA, and one qualitative study in the UK [11–26]. While anecdotal reports suggest that some UK shops offer services such as smoking cessation advice and receive referrals from local health services, there are no quantitative studies of these or other vape shop functions and activities in the UK. We now report cross-sectional surveys of the products and services, including smoking cessation advice, offered in vape shops in the East Midlands region of the UK, and of the patterns of tobacco and e-cigarette use and sociodemographic characteristics of their customers.

## Methods

### Study population

The study population included shop staff (including owners and managers) and customers in vape shops across three counties in the East Midlands region (Nottinghamshire, Lincolnshire and Leicestershire). Eligible participants were 18 years of age or older and were able to give informed consent. Using an online UK e-cigarette shop directory we identified 97 e-cigarette shops across the three counties [27]. We estimated that a sample of 59 would provide estimates of proportions (for example of shops providing cessation advice) to within 13%. We identified a random sample of 65 of the shops, stratified by county and urban or rural location, using the random sampling option in Stata 14. Our sample allowed for a 10% refusal rate.

### Data collection

#### Staff survey

Shop staff were contacted by telephone to seek their agreement for a researcher to visit their shop to collect data. Visits were undertaken on a mixture of weekdays and weekends to maximise the representativeness of the customer sample. Shop questionnaires were completed over the phone or face-to-face. All data were collected between January and April 2016.

The staff questionnaire comprised 36 questions and asked about the types of products available and product prices and promotions. The questionnaire also explored the shops’ role in smoking cessation, such as whether customers seek advice about quitting smoking in shops, whether shops provide information about quitting, whether vape shops are an appropriate environment in which to offer smoking cessation advice and how this should be implemented (for example, by a trained member of shop staff or an external trained advisor).

#### Customer survey

As not all shop staff were willing to allow the study researcher to approach customers directly, customer data were collected in two ways. In shops which permitted a direct approach, the researcher approached all customers entering the shop during the visit, explained the background to the study and asked if they would like to participate. Customers who agreed to take part completed the questionnaire face-to-face with the researcher. In shops which did not allow customers to be approached directly, the researcher left printed copies of the questionnaire for customers to complete and to be collected from the shop at a later date.

The customer questionnaire comprised 41 questions and assessed socio-demographic characteristics, patterns of e-cigarette and tobacco use, motivation for using e-cigarettes, preferences for type of e-cigarettes product, their use in smoking cessation and risk perceptions. Customers’ views about e-cigarette shops were also explored. Questions on tobacco use and previous quit attempts were obtained from the Smoking Tool Kit Study [2], and sociodemographic questions from the General Lifestyle Survey [3] and the Integrated Household Survey [4]. Customers who completed the survey were offered the opportunity to be entered into a prize draw to win to win £200, £100 or £50.

#### Piloting

The staff questionnaire was piloted in five shops. The customer questionnaire was piloted in members of the UK Centre for Tobacco and Alcohol Studies’ public involvement group, a group of Nottingham-based smokers, ex-smokers and e-cigarette users. Both questionnaires are available in online supplementary material (Additional files 1 and 2).

### Statistical analysis

All data were inputted in Excel and exported into SPSS-22 for data management and statistical analysis. Descriptive analysis was used to explore key aspects of the data. Because some of the data were obtained via self-completion and some face-to-face respondents said they had insufficient time to complete the full questionnaire, there were some missing data. These are reported in a ‘no response’ category.

## Results

### Vape shop staff survey

Staff in 41 (63%) shops agreed to participate in staff surveys, and 36 of these also agreed to allow customer interviews. The shops that declined to take part tended to be small independent shops, and in most cases did so because they were concerned that their customers would not want to be asked to complete a survey. 14 of the staff respondents were shop owners, 17 were managers and 10 were other staff members.

#### Types of e-cigarette products available

All shops stocked starter kits (which typically include a device and a bottle of e-liquid), e–liquids, atomisers, batteries and accessories (Table 1). Nicotine strengths of e–liquids ranged from 0 to 36 mg/ml. All shops stocked a 0 mg/ml nicotine e-liquid, and over half (54%) stocked 24 mg/ml liquids; 10% stocked liquids containing up to 36 mg/ml. In 17% of shops the highest nicotine concentration was 18 mg/ml, and in a further 17% it was 20 mg/ml.

**Table 1:** Vape shop products, prices and promotions

Question	*n* (%)
Highest nicotine content liquid (mg/ml)	
0	1 (2)
18	7 (17)
20	7 (17)
24	22 (54)
36	4 (10)
E-liquid bottle sizes stocked (ml, multiple responses permitted)	
1–2*	19 (46)
10	41 (100)
15	8 (20)
30	41 (100)
50–60	9 (22)
100–200	7 (17)
Flavours stocked (multiple responses permitted)	
Fruit	41 (100)
Tobacco	41 (100)
Menthol	41 (100)
Other	26 (63)
Most popular flavours (multiple responses permitted)	
Fruit	32 (78)
Tobacco	20 (49)
Menthol	27 (66)
Other	7 (17)
Cheapest starter kit price (£)	
≤ 10	4 (10)
10–19.99	19 (46)
20–29.99	15 (37)
≥ 30	2 (5)
No response	1 (2)
Most expensive starter kit price (£)	
≤ 10	0 (0)
10–19.99	3 (7)
20–29.99	3 (7)
30–49.99	6 (15)
50–99.99	17 (41)
≥ 100	10 (24)
No response	2 (5)
Promotions (multiple responses permitted)	
None	2 (5)
Buy one get one free	5 (12)
Discounts	22 (54)
Free trials	5 (12)
Other	23 (56)

Note: Percentages are rounded to the nearest whole number*E-liquid bottles usually start at 10 ml – respondents reporting this size are likely to be referring to prefilled cartridges

The most commonly stocked sizes of e-liquid bottle were 10 ml (100%) and 30 ml (100%). Every shop stocked menthol, tobacco and fruit e-liquid flavours. 63% of shops reported stocking other flavours; among those who specified these flavours, dessert-based flavours were the most common. The majority of staff (78%) reported that the fruit flavours were the most popular among their customers.

#### Price and promotions

There was a wide variety in price ranges between shops. In 46% of shops starter kits ranged from £10–£20, and in 37% they cost £21–£30 (Table 1). Promotions included discounts and free trials of starter kits. The prices of e-liquids did not increase with nicotine strength in any of the shops.

#### Smoking cessation advice and types of services available

A large proportion of staff respondents reported that customers asked for information about quitting smoking (90%; Table 2). Less than half (41%) reported providing any sort of smoking cessation advice, although 76% of staff reported feeling quite or very confident about delivering cessation advice to customers who ask for it. The majority of shops offered information about the products (93%) and cutting down on smoking (88%). Staff reported providing other information, including battery safety, operation and maintenance. The majority of staff accessed the information they gave to customers online and/or provided information based on their personal experiences.

**Table 2:** Information and support provided by vape shops (*n* = 41)

Question	*n* (%)
Do customers talk about experiences of trying to quit smoking?	
Yes many do	30 (73)
Yes some do	9 (22)
No	1 (2)
No response	1 (2)
Do customers ask for advice about quitting?	
Yes	37 (90)
No	3 (7)
No response	1 (2)
What kind of information do you give to customers? (multiple responses permitted)	
Product information	38 (93)
Smoking cessation advice	17 (41)
Information how to cut down regular cigarettes	36 (88)
Other	9 (22)
Where do you access this information? (Open question)	
Online	22 (54)
In-house training	6 (15)
Personal experience	10 (24)
Manufacturers' guidelines	3 (7)
How confident are you about offering smoking cessation advice to customers that ask for it?	
Not at all	0 (0)
Not too confident	2 (5)
Quite confident	15 (37)
Very confident	16 (39)
No response	8 (20)
Do you think it would be a good idea to deliver smoking cessation advice with training in your shop?	
Yes	19 (46)
No	4 (10)
Not sure	17 (41)
No response	1 (2)
Do you think customers would be interested in accessing this in-store smoking cessation service?	
Yes	17 (41)
No	9 (22)
Not sure	15 (37)
Which type of in-store cessation service do you think might work best? (*n* = 19 - staff who think service is a good idea)	
Support from a training member of staff	14 (74)
Face-to-face support from an external advisor	1 (5)
A text or email-based support service	0 (0)
No response	4 (21)

Note: Percentages are rounded to the nearest whole number

**Table 3:** Participants Demographic Characteristics (*n* = 197)

Question	*n* (%)
Sex	
Male	113 (57)
Female	80 (41)
No response	4 (2)
Age	
18–25	50 (25)
26–30	43 (22)
31–39	35 (18)
40–49	24 (12)
50–59	17 (9)
60+	23 (12)
No response	5 (3)
Ethnic group	
White	167 (85)
Mixed/multiple ethnic group	4 (2)
Asian/Asian British	15 (8)
Black African Caribbean/black British	2 (1)
Other ethnic group	1 (1)
No response	8 (4)
Education	
Degree level or equivalent	26 (13)
A Level, equivalent or higher education below degree level^a^	53 (27)
GCSE or equivalent^a^	52 (26)
Other below degree level	5 (3)
No formal qualifications	40 (20)
No response	21 (11)
Employment	
In full time work	111 (56)
In part time work	30 (15)
Retired	18 (9)
Student	12 (6)
Not in work (long term sick, disabled, unemployed, other reason)	19 (10)
No response	7 (4)

Note: Percentages are rounded to the nearest whole number

^a^GCSEs and A-levels are secondary school leaving qualifications in the UK. GCSEs are typically taken at age 16, A-levels at age 18

Just under half of staff respondents thought it was a good idea to deliver smoking cessation advice from a trained individual in their shops; a similar proportion were unsure. Among those who felt it was a good idea, 74% thought the best way to deliver it would be through support from a trained existing member of staff (Table 2).

### Vape shop customer survey

The customer survey was completed by 197 customers, either by self completion (83 responses) or face-to-face interview (114 responses).

#### Demographic characteristics and e-cigarette use

The majority of customer participants were male, white British, aged between 18 and 39 years, in full time employment and held a formal qualification (Table 3).

The majority of participating customers (84%) currently used e-cigarettes (Table 4). Of the 16% that did not, half (8%) were considering using an e-cigarette, a quarter (4%) were purchasing for somebody else and a further quarter (4%) were collecting information only. Among participants currently using an e-cigarette, 19% were dual users (e-cigarettes and regular cigarettes) and 78% had quit smoking cigarettes. A minority (3%) of those using e-cigarettes had never smoked cigarettes.

**Table 4:** Tobacco use, e-cigarette use and smoking cessation

Question	*n* (%)
All participants (*n* = 197)	
Reason for vape shop visit	
I am currently using e-cigarettes	165 (84)
I am considering using e-cigarettes	15 (8)
I am purchasing e-cigarettes for somebody else	8 (4)
I am visiting to collect information	8 (4)
Other	1 (1)
All e-cigarette users (*n* = 165)	
Tobacco and e-cigarette use	
I currently use both e-cigarettes and regular cigarettes	31 (19)
I currently use e-cigarettes only but used to smoke regular cigarettes	128 (78)
I currently use e-cigarettes but have never smoked regular cigarettes	5 (3)
No response	1 (1)
Time since starting e-cigarette use	
Less than 24 h	4 (2)
Between 1 day and 1 week	1 (1)
Between 1 week and 4 weeks	15 (9)
Between 1 month and 1 year	47 (28)
Between 1 year and 2 years	56 (34)
Longer than 2 years	41 (25)
Don’t know/other	1 (1)
Reasons for starting to use an e-cigarette (multiple responses permitted)	
Wanted to quit smoking	129 (78)
Previous unsuccessful attempt	33 (20)
Reduce number I smoke	26 (16)
Friends/family	40 (24)
Flavours I like	24 (15)
Advert	1 (1)
Can use e-cigarette where can’t smoke	29 (18)
E-cigarettes are less harmful	55 (33)
Cheaper than regular tobacco	53 (32)
Curiosity	24 (15)
Other	7 (4)
Main reason for using an e-cigarette	
To help me quit/stay quit	117 (71)
To reduce number of regular cigarettes I smoke	17 (10)
I can use e-cigarettes where I can’t smoke	6 (4)
Other	12 (7)
No response	13 (8)
Perceived harm of using e-cigarette	
More harmful than tobacco cigarettes	3 (2)
Less harmful than tobacco cigarettes	136 (82)
The same as tobacco cigarettes	2 (1)
Don’t know	17 (10)
No response	7 (4)
Side effects while using e-cigarette	
No side effects	120 (73)
Side effects	45 (27)
Weekly spend on e-cigarettes	
< 5£	38 (23)
£5–10	63 (38)
£11–20	33 (20)
£21–30	13 (8)
Other	7 (4)
Don’t know	3 (2)
No response	8 (5)
Nicotine content used when started vaping (mg/ml)	
0	3 (2)
1–3	5 (3)
4–10	16 (10)
11–20	74 (45)
> 20	43 (26)
No response	24 (15)
Nicotine concentration currently used (mg/ml)	
0	12 (7)
1–3	52 (32)
4–10	41 (25)
11–20	33 (20)
> 20	10 (6)
No response	17 (10)
Change in nicotine content since started vaping	
Reduced	92 (56)
Stayed the same	41 (25)
Increased	0 (0)
No response	32 (19)
Preferred e-liquid flavour	
Fruit	80 (48)
Tobacco	19 (12)
Menthol	21 (13)
Other	19 (12)
No response	23 (14)
Where usually buy e-cigarettes or liquids	
From a shop	117 (71)
Online	2 (1)
Both	35 (21)
Other	2 (1)
No response	9 (5)
Type of information/advice received from shop (multiple responses permitted)	
About the products	148 (90)
About stopping smoking tobacco cigarettes	62 (38)
About how to cut down on smoking tobacco cigarettes	46 (28)
Other	2 (1)
Appropriate to deliver advice on quitting smoking in an e-cigarette shop?	
Yes and I would consider using it	75 (45)
Yes but I would not consider using it	5 (3)
Maybe	40 (27)
Not sure	15 (9)
No	10 (6)
Don’t want to answer	5 (3)
No response	15 (9)
Best way to deliver in-shop service (*n* = 75 – vapers who would consider using service)	
Support from a trained member of staff	58 (77)
Face-to-face support from an external advisor	2 (3)
A text or email-based support service	7 (9)
Other	1 (1)
No response	7 (9)
Ex-smokers only (*n* = 128)	*n*
Time since quit smoking	
Within the last week	5 (4)
Within the last month	8 (6)
Within the last 6 months	14 (11)
Within the last year	24 (19)
Over a year ago	75 (59)
No response	2 (2)
Methods used to help stop smoking during the most recent serious quit attempt (multiple responses permitted)	
Electronic cigarette	114 (89)
Nicotine replacement therapy	48 (38)
Nicotine replacement on prescription	12 (9)
Zyban	5 (4)
Champix	14 (11)
Stop smoking group	11 (9)
Smoking helpline	4 (3)
NHS smoke free website	9 (7)
App	5 (4)
Hypnotherapy	3 (2)
Acupuncture	2 (2)
Booklet	1 (1)
Don’t know	9 (7)
Other	2 (2)

Note: Percentages are rounded to the nearest whole number

The main reasons reported for taking up vaping were wanting to quit smoking, the belief that vaping is less harmful than smoking, and e-cigarettes being cheaper than regular tobacco. The majority of e-cigarette users, 86%, perceived EC to be less harmful than regular cigarettes, and 82% had experienced no side effects from vaping. Among those who had, the side effects headaches, chest pain/shortness of breath, coughs and colds and dry mouth/throat were the most frequently reported. Most vapers (64%) reported spending up to £10 a week on e-cigarettes.

More than half of participants reported using a lower level of nicotine in their current e-liquid than when they first started using an e-cigarette (Table 4). The majority (57%) reported currently using an e-liquid containing 1-10 mg/ml, and a small proportion used zero nicotine liquids.

#### Ex-smokers

The majority of ex-smokers reported having quit more than one year ago (59%). Health concerns and the costs of smoking were frequently cited reasons for quitting. Most (89%) had used an e-cigarette to help them quit, but two in five had also used nicotine replacement therapy.

#### Dual users

There was a small proportion of dual users in the sample (*n* = 31, 19%). 65% of dual users reported smoking tobacco cigarettes every day, while 61% also reported smoking a tobacco cigarette within the last 24 h. However, 58% intended to give up tobacco smoking within the next 6 months. Half of dual users stated their most recent quit attempt was expense-related (52%) and due to a future health concern (45%). (Data not shown).

#### Vape shop experiences and smoking cessation services

Nearly three quarters of participants who reported vaping (71%) said they usually buy their vaping products from a shop. Most participants reported receiving information about the products from the shop they were in. Around two in five said they received information on quitting and just over a quarter received information about cutting down. Just under half (45%) stated that they thought it was appropriate to deliver smoking cessation advice and support in the shop and that they would consider using such support; of these, three quarters felt that the best way to deliver such a service was through a trained member of staff.

## Discussion

This study found that vape shops cater for customers seeking a wide range of nicotine content, flavours and price points, and offer a variety of promotions. Most vape shop customers were current vapers who had quit smoking, and were using an e-cigarette to either quit or stay quit. A very small minority were vapers who had never smoked. The majority of customers had reduced the nicotine content of their e-liquid since taking up vaping. Shop staff reported that most customers ask for advice about quitting smoking, but that most of the information they provided was about the products they sell. There were mixed views among both staff and customers as to whether it would be appropriate to deliver smoking cessation advice in-store; however there was broad consensus that if such support were to be delivered, support from a trained member of existing staff would be preferred.

Our study has a number of strengths and limitations. Most existing vape shop studies have used qualitative techniques; by using a survey we were able to quantify key characteristics in our population, such as dual use, albeit that we were not able to analyse smoking/vaping trajectories over time. Nevertheless, some aspects, such as understanding the type and accuracy of advice given to customers, would have benefitted from qualitative interviews with shop staff.

While we only used one directory to identify shops, the use of a large vape shop-specific directory rather than a generic business directory (e.g. Yell) means that we are likely to have identified the majority of shops in our study area. Recruitment to the study was more challenging than anticipated; we approached 65 shops, but only 41 agreed to take part. While the sample size of both surveys is therefore relatively small, and prevents us from reliably analysing subgroups (e.g. new vs. long-term vapers), our findings provide an initial insight into who accesses vape shops and why, the products and services offered and the potential for capitalising on opportunities to promote smoking cessation in the vape shop setting. We used a systematic approach to identify vape shops in the East Midlands region, and a sampling strategy which maximises the representativeness of our sample. The study was conducted in a limited geographic region; however we sampled shops in both urban and rural areas to maximise the representativeness of our sample. Our study was conducted prior to the implementation of the EU Tobacco Products Directive, which imposed limits on nicotine strength (20 mg/ml) and e-liquid bottle sizes (10 ml) in the EU [28]; the typical nicotine concentration and bottles sizes will therefore have changed in UK vape shops since our study was conducted.

Our pragmatic approach to data collection means that our customer sample is not random. Some respondents, particularly customers, often lacked the time to fully complete the survey resulting in some missing data. Furthermore, we found that the vape shop setting made it impractical to collect carbon monoxide readings to confirm smoking status, and this measure is therefore based on self-report. There is a risk of social desirability bias whereby respondents are more likely say they have quit smoking cigarettes because they are in a vape shop; however, in existing research biochemical validation found that the vast majority of self-reported quitters in vape shops had indeed quit [25]. Our combination of face-to-face interviews and allowing customers to complete questionnaire in their own time is likely to have maximised the amount and quality of data collected.

Our finding that the majority of vape shop customers were ex-smokers and used e-cigarettes to stop smoking or stay quit conflicts somewhat with population-level data suggesting that around half of adults who use e-cigarettes are dual users [7, 29]. It seems likely that vapers who use specialist vape shops are not representative of vapers in general, and may represent a group more committed to vaping (and hence more likely to have quit smoking) than casual purchasers of e-cigarettes from convenience stores and supermarkets. While we are unable to draw any causal links between this population’s e-cigarette use and quitting smoking, our findings underline that many vapers are successful quitters. Furthermore, our study found that the majority of ex-smokers had quit smoking more than a year ago, suggesting that e-cigarettes may help in sustained as well as in initial cessation. While there is limited evidence as to the effectiveness of e-cigarettes for quitting when bought in vape shops, one pilot study found that at 12 month follow-up, 40% of smokers making their first purchase at a vape shop had quit smoking [16].

Around two thirds of vapers in our sample reported having cut down their nicotine concentration in their e-liquid since taking up smoking; this is consistent with a previous study conducted in the US [20]. This finding may reflect improvements in the ability of e-cigarette devices to deliver nicotine [25]; however, the level by which customers report cutting down (an average of 13 mg/ml among those reducing) suggests that e-cigarettes may enable a two-stage quitting process whereby smokers reduce or overcome their nicotine addiction before attempting to end the habitual behaviours involved and associated with smoking. However, while the majority of our sample used a relatively low nicotine concentration when they started vaping, over a quarter reported using an e-liquid containing a level of nicotine exceeding restrictions imposed by the new EU Tobacco Products Directive (20 mg/ml) [28]. This raises concerns that at least some potential vapers will be put off by fears that e-cigarettes will, at least at the outset, provide insufficient levels of nicotine.

Many customer survey respondents reported cost as a factor which influenced their decision to use e-cigarettes, and most reported a weekly spend of less than £10, i.e. less than the cost of a single pack of 20 premium cigarettes. Existing evidence suggests that 1 ml of e-liquid is consumed over a length of time equal to that which in which a typical smoker consumes 5.63 combustible cigarettes [30]. Based on this assumption, a starter kit costing £20 containing 10 ml of e-liquid would provide the equivalent of 56 cigarettes (2.8 packs of 20). The weighted average price for 20 cigarettes in the UK is currently £7.8 [31] – this suggests that, in the UK, where the price of combustible tobacco is high, the start-up cost of vaping may be similar to the cost of (licit) tobacco; the cost of ongoing e-cigarette use is likely to be lower. Furthermore, we found that shops frequently run promotions, which is likely to help keep the cost down. Smoking imposes a substantial financial burden on smokers, a high proportion of whom are already living on low incomes [32]. The lower cost of e-cigarettes and the price-based marketing approaches used by vape shop customers are therefore encouraging, as they highlight the potential of e-cigarettes as not only a less harmful but also a cheaper alternative to smoking. Existing evidence suggests that in England, vapers are more likely to be from high socioeconomic groups [7]. Ensuring that deprived smokers are informed of the financial benefits, as well as potential health benefits of switching to e-cigarettes may be a way of encouraging use in this population.

Our findings support existing evidence that e-cigarette users are more likely to have accurate perceptions of harm of vaping than the general population or smokers who do not vape; nearly all our customers respondents (82%) believed vaping to be less harmful than tobacco smoking [29]. Given that role that vape shop staff are likely to play in influencing vapers’ and potential vapers’ perceptions of harm and other aspects of vaping (such as effectiveness for smoking cessation), it is essential that they look to accurate and up-to-date sources of information about e-cigarettes. However, previous research suggests that information conveyed to vape shop customers may be incomplete or misinterpreted [13]. Our findings that staff access information in a wide range of ways, including online and based on personal experience, suggests there is a risk that staff may provide inaccurate or biased information. Future research should consider ways to ensure that vape shop customers, particularly those considering vaping for the first time, are given up-to-date evidence-based information.

While the majority of shop staff said customers ask for advice about quitting smoking and felt confident about giving such advice, less than half said they actually provide it. It is not clear whether this means that staff sometimes avoid giving advice, preferring, for example, to refer customers to a health professional; and if so, why this may be. Detailed data on the nature of advice given were not collected, and future research should seek to better understand the types of information customers receive, how that information is interpreted, and whether it is acted upon; however, the most frequent response to an open-ended question about the type of cessation advice given was ‘non-medical advice’ (*n* = 17), suggesting that shop staff may be unwilling to give clinical cessation advice. However, nine in ten staff said that they provide advice on cutting down regular cigarettes. Taken together, these findings indicate that smoking cessation and reduction are part of the dialogue between vape shop staff and customers, and suggests that there is scope for increasing the level of information vape shop customers receive about quitting using e-cigarettes.

There were mixed views about whether delivering formal smoking cessation advice in shops was a good idea. This may reflect existing evidence which suggests that to some e-cigarette users pleasure and enjoyment are central to the vaping experience, while others regard e-cigarettes as a medical treatment for nicotine addiction, with enjoyment and culture playing much less of a role [33]. A recent vape shop study by Ward et al. identified a divide between groups of e-cigarette users, with some liking the non-medicalised environment of vape shops, but others, who perceive e-cigarettes as a medical treatment, sometimes being intimidated by the vape shop setting. It has been suggested that providing ‘recreational’ and medical pathways to e-cigarette use may maximise the potential for harm reduction [33, 34], and Ward et al. suggest that ‘informal co-working between shops and stop smoking services’ could encourage smokers who are unsure about e-cigarettes [26]. Our mixed findings indicate that achieving this successfully within the vape shop setting might be challenging, given the need to appeal to those seeking a ‘medical’ solution, without putting off vapers who are not seeking ‘treatment’. However, it seems likely that some smokers may feel more confident about accessing vape shops and using e-cigarettes if they know that evidence-based support is available, and therefore that providing support may enhance vape shop profitability.

Among staff and customers who were supportive of an in-store smoking cessation intervention, most felt that the best way to deliver this type of service would be via a trained member of existing staff, although our data did not provide sufficient granularity to gain a comprehensive understanding of the type of advice and mode of delivery that might be appropriate and acceptable. Whether offering help to smokers to transition more effectively from tobacco to electronic cigarettes represents a commercial opportunity to vape shops, by helping to generate new customers; and whether this same commercial interest might then inhibit the promotion of complete cessation of vaping, remains to be seen.

## Conclusions

The majority of vape shop customers are vapers who have quit smoking. Vape shop staff play a central role in providing customers with product information, and many provide smoking cessation advice. There is a need for clear and easily accessible information on the health effects and effectiveness of e-cigarettes for smoking cessation, to ensure that vape shop staff provide customers with accurate information on their products. Further research is needed to investigate the potential for smoking cessation interventions in vape shops, including the extent to which this type of intervention would appeal to non-vapers and how and by whom it would best be delivered.

## Additional files

Additional file 1: Shop questionnaire. (DOC 321 kb)

Additional file 2: Customer questionnaire. (DOC 413 kb)
