# Supplementary material for: Vape shops: who uses them and what do they do?
Source: BMC Public Health. 2018 Apr 23;18:541. doi: 10.1186/s12889-018-5467-9 (PMC5914011; doi:10.1186/s12889-018-5467-9)
Supplement: Supplementary file 1 — Shop questionnaire. (DOC 321 kb) [file 12889_2018_5467_MOESM1_ESM.doc]

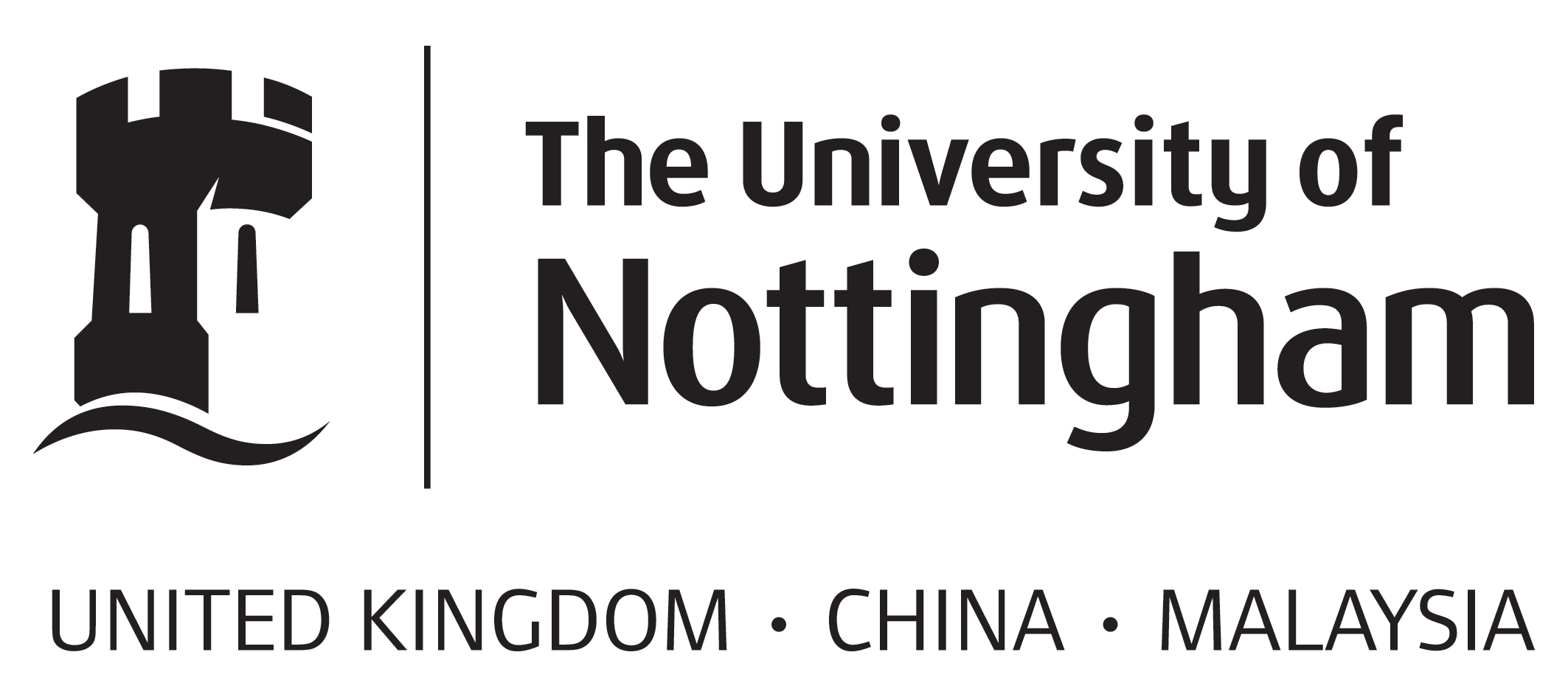

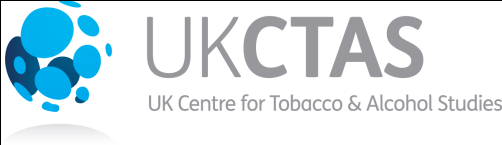


**AN EXPLORATION OF ELECTRONIC CIGARETTE SHOPS AND USERS IN THE EAST MIDLANDS**

**SHOP QUESTIONNAIRE**

This is a study collecting data on e –cigarette usage in the East Midlands. At present limited information is available about UK e-cigarette shops including products, services provided and their customers.

I am a researcher from the University of Nottingham and this study is in collaboration with Cancer Research UK. You are being invited to participate because you an e –cigarette provider. By participating in this study you will help create clearer understandings about e-cigarette usage which may help you improve services you provide to your customers. The researcher will also ask your permission to ask some of your customers to complete a short questionnaire lasting 5 – 10 minutes. The researcher will provide you with all the details about the survey and will collect the data in a discreet manner.

The researcher will talk over and provide you with an information sheet to help you understand why the research is being conducted and how it will involve you. The survey will take approximately 5 -10 minutes to complete.

As appreciation for your time at the end of the shop questionnaire, you will be offered the opportunity to be entered into a draw for a prize of £100.

I have read the information sheet and I agree to participate in this survey only

I agree to participate in this survey and for the researcher to survey customers

**Section 1: Background**

1. I am the shop?

- Owner
- Manager
- Other staff member

1. How long have you been in operation?

- Months
- Years

1. Which days are you open? (Please tick all that apply)

- Monday
- Tuesday
- Wednesday
- Thursday
- Friday
- Saturday
- Sunday

1. Which is your busiest day?

- Monday
- Tuesday
- Wednesday
- Thursday
- Friday
- Saturday
- Sunday

1. On a typical day, what is your busiest time period?

- 9:00am-12:00pm
- 12:00pm-3:00pm
- 3:00pm-6:00pm
- 6:00pm-9:00pm
- Other

1. On average, how many customers do you have each day?

- Less than 10
- 11- 30
- 31-50
- 51-100
- 101 +

1. On average, how many customers do you have on your busiest day of the week?

- Less than 10
- 11- 30
- 31-50
- 51-100
- 101 +

**Section 2 Products**

1. What are the ranges of available products? e.g. e-liquids, atomisers, batteries

………………………………………………………………………………………………………………………………………………………………………………………………………………………………………………………………………………………………………….

1. What is the price of the cheapest starter kit that you stock?

……………………………………………………………………………………………………..

3. What is the price of the most expensive starter kit that you stock?

……………………………………………………………………………………………………

4. Which e-liquid volumes do you have available? (Tick all that apply)

- 10ml
- 30ml
- Sample packs
- Other (please specify)

5. E-liquid flavours available (Tick all that apply)

- Fruit
- Tobacco
- Menthol
- Other (please specify)

6. What are the most popular e-liquids? (Tick all that apply)

- Fruit
- Tobacco
- Menthol
- Other (please specify)

1. What is the nicotine concentration of the;
2. lowest nicotine concentration liquid that you sell?..................
3. highest nicotine concentration liquid that you sell?.....................
4. Do prices of liquids vary according to nicotine concentration?

- Yes – higher nicotine concentrations are more expensive
- Yes – lower nicotine concentrations are more expensive
- No
- Don’t know

1. Types of atomisers (Tick all that apply)

- Dual coil
- Single coil
- Others (please specify)

1. What is the price of?

i) the cheapest atomiser that you sell……………………………..

ii) the most expensive atomiser that you sell…………………

11. What is the price ranges of the batteries that you sell? (Tick all that apply)

- £1-£5
- £6-£10
- £11-£20
- £21-30
- Other

12. What type of promotions do you offer?

- No promotions
  - Buy one get one free
  - Discount
  - Free trials using products
  - Others (please specify

13. Could you please state your most popular brand/s?......................................................

**CUSTOMER SERVICE**

1. On average, how often do your customers return to the shop?

- Daily
- Weekly
- Fortnightly
- Monthly
- Less often
- I don’t know

1. Why do you think customers choose your shop over others?

(Please tick All that apply)

- - Fair prices
  - Close to where I shop
  - Wide range of products on offer
  - Unique flavors / hardware
  - Wide range of nicotine content
  - Relaxed atmosphere
  - Friendly and helpful staff
  - Quick service
  - Online store capability
  - Clean environment
  - Community
  - Other (Please specify)

1. Has there been an increase in the average number of customers over the past year and by how much? (For those who have been operating for more than one year)
   - No increase
   - 10%
   - 20%
   - 50%
   - 70%
   - 100%
   - Other
2. When customers visit the shop, do they talk about their experiences of trying to quit smoking?

- Yes, many do
- Yes, some do
- No

1. Do customers ask for advice about quitting smoking?

- Yes
- No

1. What kind of information do you give to customers? (Tick all that apply)

- Product information
- Smoking cessation advice
- Information on how to cut down on regular cigarettes
- Other (please specify)

7. Where do you access this information that you provide to customers? (Open question) ……………………………………………………………………………………………………………………………………….…………

………………………………………………………………………………………………………………………………………………………………………………………………………………………………………………………………………………………………………………………….

*If you offer smoking cessation advice, go to question 8, if not go to question 10.*

8. What smoking cessation advice do you give? (Open-ended question)

…………………………………………………………………………………………………………………………………………………………………………………………………………………………………………………………………………………………………………………………………………………………………………………………………………………………………………………………………………..

9. How confident are you about offering smoking cessation advice to customers that ask for it?

- Not at all confident
- Not too confident
- Quite confident
- Very confident

10. Do you think it would be a good idea to offer smoking cessation support with relevant training in your shop to customers interested in either quitting cigarette smoking or cutting down?

- Yes
- No
- Not sure

11. Do you think customers would be interested in a smoking cessation intervention delivered via your shop?

- Yes
- No
- Not sure

12. If yes, which intervention do you think might work best?

- Support from a trained member of staff already working in the shop
- Face-to-face support from an external advisor (e.g. from local stop smoking service)
- A text or email based support service
- Other (please specify)

13. What are some of the common concerns expressed by customers?

- The harmfulness of e-cigarettes
- The harmfulness of e-cigarettes compared with tobacco cigarettes
- Side effects of e-cigarettes
- Others (please specify)

14. Do you get any referrals from the NHS?

- No
- Yes

15. If yes, who from?

- GPs
- Nurses
- Pharmacist
- Others (please specify)

1. Would you be interested in taking part in future research?

- Yes
- No

Thank you for completing this survey

----------------------------------------------- To be detached for the prize draw-------------

Staff name…………………………………………………………………………………………………………………

Phone number…………………………..……..…………E.Mail………………………………………………………
