# Supplementary material for: Vape shops: who uses them and what do they do?
Source: BMC Public Health. 2018 Apr 23;18:541. doi: 10.1186/s12889-018-5467-9 (PMC5914011; doi:10.1186/s12889-018-5467-9)
Supplement: Supplementary file 2 — Customer questionnaire. (DOC 413 kb) [file 12889_2018_5467_MOESM2_ESM.doc]

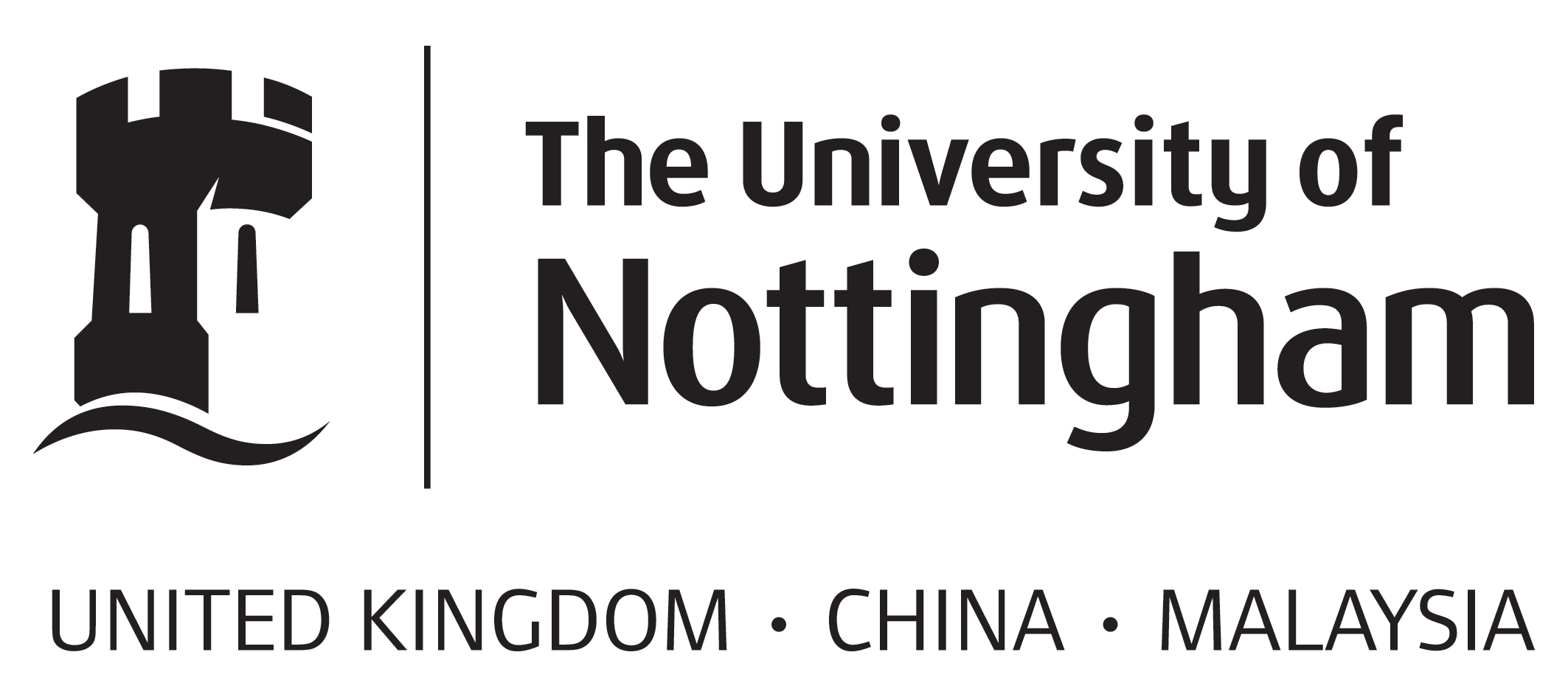

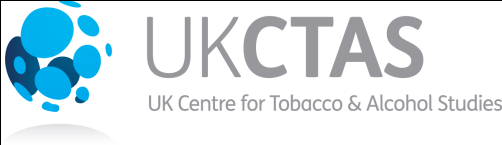


**ELECTRONIC CIGARETTE SHOPS AND USERS IN THE EAST MIDLANDS:**

**CUSTOMER SURVEY**

This survey is part of a study collecting data on e–cigarette usage in the East Midlands. At present limited information is available about UK e-cigarette shops including products, services provided and their customers.

The study is being carried out by researchers from the University of Nottingham and is funded by Cancer Research UK. By participating in this study you will give us a better understanding of e-cigarette use.

The survey will take approximately 5-10 minutes to complete. To show appreciation for your time, we are offering you the chance to be entered into a prize draw to win a 200, £100 or £50 High Street Shopping Voucher. All information provided is confidential and anonymous. Participation is voluntary and you are free to withdraw from the survey at any stage.

The researcher will provide you with an information sheet before you decide to take part. At the end of the survey you will be asked to measure your CO (carbon monoxide) levels by blowing into a CO monitor, which is completely voluntary. You may still choose to complete this survey, even if you decide not to provide a CO measure. If you have any questions, please ask the researcher.

**Please tick the relevant box**

Survey completed by researcher Survey not completed by researcher

**What is the purpose of your visit today?**

I am considering using e –cigarettes go to **Section 1**

I am a currently using e –cigarettes go to **Section 2**

I am purchasing e- cigarettes for somebody else go to **Section 6**

I am visiting to collect information only go to **Section 6**

Other go to **Section 6**

**Section 1.**

**1. What has prompted you to consider using e-cigarettes?** Please tick all the following that apply

| - Friends/Family - An advertisement - Curiosity - Less harmful than regular cigarettes | - I can use e-cigs where I can’t smoke - To reduce the number of regular smoked - I would like to quit smoking - Previous unsuccessful attempts - Cheaper than smoking regular cigarettes |
| --- | --- |

**2. When comparing e-cigarettes with tobacco cigarettes, do you think e-cigarettes are?** Please tick one from the below

| - More harmful than tobacco cigarettes - Less harmful than tobacco cigarettes | - The same as smoking tobacco cigarettes - Don’t know |
| --- | --- |

1. **What do you think may be the disadvantages of e-cigarettes?** Please tick all that apply.

| - None - May not be safe | - May make it harder to quit - May not satisfy the desire to smoke |
| --- | --- |

**Section 2. About Smoking** Which of the following best applies to you?

| I currently use both e-cigarettes and regular cigarettes |  |  | go to question 1below |
| --- | --- | --- | --- |
| I currently use e-cigarettes only but I used to smoke  regular cigarettes |  |  | go to Section 3 |
| I currently use e-cigarettes but have never smoked regular cigarettes |  |  | go to Section 4 |

1. Which of the following best applies to you?

| - I smoke cigarettes (including hand-rolled) every day - I do not smoke cigarettes at all, but I do smoke tobacco of some kind (e.g. pipe or cigar) everyday | - I do not smoke cigarettes at all, but I do smoke tobacco of some kind (e.g. pipe or cigar) but not everyday - Don’t know |
| --- | --- |

1. On average, how many cigarettes did you smoke before starting to use e-cigarettes?

Each day Each Week

1. On average, how many cigarettes do you smoke now?

Each day Each week

1. About how long has it been since you smoked a regular cigarette?

| - Less than 24 hours - Between 1 day and 1 week - Between 1 week and 4 weeks - Between 4 weeks and 12 weeks | - Between 12 weeks and 6 months - Longer than 6 months - Don’t know |
| --- | --- |

1. Which of the following statements best describes you?

| - I intend to give up smoking within the next 6 months - I intend to give up within the next year - I intend to give up smoking but not in the next year | - I intend to give up smoking but I am not sure when - I don’t intend to give up smoking - Don’t know |
| --- | --- |

1. How many serious attempts to stop smoking have you made in the last 12 months?

By serious attempt I mean you decided that you would try to make sure you never smoked again. Please include any attempt that you are currently making and please include any successful attempt made within the last year…………

1. Which of the following do you think contributed to you making the most recent quit attempt? Please tick all that apply

| - Advice from a GP/health professional - TV advert for nicotine replacement product - Government TV/radio advert - Seeing a health warning on a cigarette packet - A concern about my future health - Something friend/children said | - Hearing about a new stop smoking treatment - Decided smoking was too expensive - Being faced with smoking restrictions - I knew somebody else was stopping - Being contacted by my local NHS stop smoking service - Health problem at the time - Attending a local stop smoking group - Significant birthday - Other |
| --- | --- |

1. Which, if any, of the following did you use to try to help you stop smoking during the most recent serious quit attempt? Please tick ALL that apply

| - Electronic cigarette - nicotine replacement product e.g. patches gum without prescription - nicotine replacement on prescription - Zyban - Champix - Attended a stop smoking group - Phoned smoking Helpline | - Visited NHS stop smoke free website - Used an app (e.g. smartphone) - Hypnotherapy - Acupuncture - Booklet/visited non UK website - Don’t know - Other   ***🡪 Please go straight to Section 4.*** |
| --- | --- |

**Section 3. About quitting (ex-smokers only)**

| 1. How long ago did you quit smoking?  - Within the last week - Within the last month - Within the last 6 months | - Within the last year - Over a year ago |
| --- | --- |

1. Which of the following do you think contributed to you making the most recent quit attempt? Please tick all that apply

| - Advice from a GP/health professional - TV advert for nicotine replacement product - Government TV/radio advert - Seeing a health warning on a cigarette packet - Concern of future health problem - Something friend/children said - A significant birthday | - Hearing about a new stop smoking treatment - Decided smoking was too expensive - Being faced with smoking restrictions - I knew somebody else was stopping - Being contacted by my local NHS stop smoking service - A health problem at the time - Attending a local stop smoking activity - Other |
| --- | --- |

1. Which, if any, of the following did you try to help you stop smoking?

| - Electronic cigarette - nicotine replacement product e.g. patches gum without prescription - nicotine replacement on prescription - Zyban - Champix - Attended a stop smoking group - Phoned smoking Helpline | - Visited NHS stop smoke free website - Used an app (e.g. smartphone) - Hypnotherapy - Acupuncture - Don’t know - Other - Booklet/visited non UK website   ***🡪 Please go straight to Section 4.*** |
| --- | --- |

**Section 4. About E-cigarettes**

1. How long has it been since you started using e-cigarettes?

| - Less than 24 hours - Between 1 day and 1 week - Between 1 week and 4 weeks - Between 6 months and 1 year | - Between 1 year and 2 years - Longer than 2 years - Don’t know - Other |
| --- | --- |

1. What made you start using e-cigarettes? (Tick all that apply)

| - Wanted to quick smoking - Previous unsuccessful attempt - Reduce the number of regular cigarettes I smoke - Friends/family - Flavors I like - Advertising | - I can use e cigs where I can’t smoke - E-cigs are less harmful - Cheaper than smoking regular cigarettes - Curiosity - Don’t know - Other |
| --- | --- |

1. What is your **main** reason for using e-cigarettes?

| - To help me quit smoking/stay quit - To reduce the number of regular cigarettes I smoke | - I can use e cigs where I can’t smoke - Other |
| --- | --- |

1. Do any of the following apply to you?

| - I socialise and feel a sense of community with other e cig users - I use e cigarette forums | - I attend vaping events e.g. festivals |
| --- | --- |

1. On average, how many times per day do you use an e-cigarette?

| - 0 - 1-5 - 6-10 | - 11-15 - 16-20 - 21+ |
| --- | --- |

1. On average, what is your daily consumption of e-cigarettes?

| - Ml of liquid/ day……………. | - No. of cartridges/day…………. |
| --- | --- |

1. What was/is?

| 1. the nicotine content of your e-liquid when you first started using an e cigarette? | b) The content of the e liquid you are currently using for your e-cigarette? |
| --- | --- |

1. Which is your preferred e-liquid flavor?

| - Fruit - Menthol | - Tobacco - Other |
| --- | --- |

1. On average how much do you think you spend on e-cigarettes each week?

| - Less than £5 - £5-10 - £ 11-20 | - £21-30 - Other - Don’t know |
| --- | --- |

1. How soon after you wake up do you light up an e-cigarette?

| - Within 5 mins - 6-30 mins - 31-60 mins | - Greater than 60 mins - Other - Don’t know |
| --- | --- |

1. When comparing e-cigarettes with tobacco cigarettes, do you think e-cigarettes are?

| - More harmful than tobacco cigarettes - Less harmful than tobacco cigarettes | - The same as smoking tobacco cigarettes - Don’t know |
| --- | --- |

1. What do you believe to be the disadvantages of e-cigarettes? Please tick all that apply.

| - None - May not be safe - May make it harder to quit | - May encourage the uptake of using tobacco - Does not satisfy the desire to smoke |
| --- | --- |

1. Have you noticed any side effects associated with e-cigarettes and if so what are they?

……………………………………………………………………………………………………………………………………………………………………………………………………………………………………………………………………………………………………………………………………………………………………………………….

1. Where do you usually buy your e-cigarettes or e-cigarette liquids?

| - From a shop - Online | - Both - Other please specify………………… |
| --- | --- |

**Section 5. About this shop**

1. How often do you visit this shop?

| - Daily - Weekly - Fortnightly | - Monthly - It is my first visit - Other |
| --- | --- |

1. How long have you been using this e-cigarette shop?

| - It is my first visit - A couple of weeks - 1-2 months - 3-4 months - 5-6months | - 6-12 months - 1-2 years - 2 – 3 years - 3 years or more |
| --- | --- |

1. Have you visited other e-cigarette shops?

| - Yes | - No |
| --- | --- |

1. Why did you decide to use this particular shop? Please tick all that apply

| - Fair Prices - It’s the nearest one to where I live - It’s the nearest one to where I work - Wide range of nicotine content - Relaxed atmosphere - Clean environment | - I shop in the area - Unique flavors – hardware - Online store capacity - Wide range of products on offer - Friendly and helpful staff - Quick service - Other please specify……………….. |
| --- | --- |

1. What kind of information/advice do you get from this shop? Please tick all that apply?

| - About the products - About stopping smoking | - About how to cut down on smoking - Other please specify……. |
| --- | --- |

1. Do you think it would be appropriate to deliver advice and support for quitting smoking in this shop?

| - Yes, and I would consider using it - No - Don’t want to answer | - Yes, but I would not consider using it - Maybe - Not sure |
| --- | --- |

1. If yes, which method would you most prefer?

| - Support from a trained member of staff - A text or e mail based service | - Face to face support from an external advisor - Other please specify……. |
| --- | --- |

**Section 6. Demographics**

| 1. Sex  - Male | - Female |
| --- | --- |

1. **Age category**

| - 18-25 - 31-39 - 50-59 | - 26-30 - 40-49 - 60+ |
| --- | --- |

1. What is your ethnic group? Choose one that best describes your ethnic group or background.

| - White - Mixed/multiple ethnic group - Asian/Asian British | - Black African Caribbean/black British - Other ethnic group |
| --- | --- |

1. Which of these categories best describes you at present?

| - Working part time as an employee - Working full time as an employee - Working full time self employed - Working part time self employed - Long term sick or disabled - Retired from paid work | - Unemployed - Student - Student including pupil at school, or in training - Looking after family home - Not in paid work for some other reason |
| --- | --- |

1. What is the highest educational qualification level you have?

| - Degree level qualification or equivalent - Higher education below degree level - A levels/Higher/ Advanced diploma/ progression diploma - ONC /National Level BTEC - No formal qualifications | - O-Level or GCSE equivalent (grade A-C) or O Grade/CSE equivalent (Grade 1) or Standard Grade Level 1 -3 or Higher Diploma - GCSE grade D-G or CSE grade 2-5 or Standard Grade level 4-6 or Foundation Diploma - Other qualifications (including foreign qualifications below degree level) |
| --- | --- |

1. How many children under 18 live in your household?……………….
2. How many children under 5 live in your household?....................

**Measuring carbon monoxide levels**

Please provide a measure of your CO (carbon monoxide) levels by blowing into the CO monitor.

- Yes, I agree and my CO result is
- I would prefer not to give a measure

**Thank you for taking the time to complete this survey**

-----------------------------------------------------------------------To be detached----------------------------------

**Prize draw contact details for £200, £100 or £50 shopping Voucher**

If you would like to be included in the prize draw please provide your details below.

Name……………………………………………………………………………

E- mail/phone number …………………………………………………………………………………………….

**Opportunity to participate in further research**

The researcher may contact you at a later stage to take part in a short interview lasting approximately 20 minutes over the phone to gain a deeper understanding about your vaping experiences. You will be asked to complete a consent form today if you would like to participate. To show appreciation you will automatically be posted a £10 shopping voucher after the interview.
